# Hydroxonium creatininium bis­(pyridine-2,6-dicarboxyl­ato-κ^3^ *O* ^2^,*N*,*O* ^6^)nickel­ate(II) trihydrate

**DOI:** 10.1107/S1600536809022053

**Published:** 2009-06-27

**Authors:** Jafar Attar Gharamaleki, Hossein Aghabozorg, Zohreh Derikvand, Mohammad Yousefi

**Affiliations:** a Young Researchers Club, Islamic Azad University, North Tehran Branch, Tehran, Iran; b Faculty of Chemistry, Islamic Azad University, North Tehran Branch, Tehran, Iran; c Department of Chemistry, Faculty of Science, Islamic Azad University, Khorramabad Branch, Khorramabad, Iran; d Department of Chemistry, Islamic Azad University, Shahr-e Rey Branch, Tehran, Iran

## Abstract

The title compound, (C_4_H_8_N_3_O)(H_3_O)[Ni(C_7_H_3_NO_4_)_2_]·3H_2_O, exhibits isotypism with its Co^II^ analogue. All intramolecular distances and angles are similar for the two structures. This applies also for the intermolecular forces, consisting of O—H⋯O and N—H⋯O hydrogen bonds and π–π interactions [with centroid–centroid distances of 3.428 (2) and 3.579 (2) Å], that lead to a cohesion of the structure.

## Related literature

For background to proton-transfer agents, see: Aghabozorg, Manteghi *et al.* (2008 ▶); Soleimannejad *et al.* (2005 ▶); Aghabozorg, Ramezanipour *et al.* (2008 ▶). For related structures, see: Moghimi *et al.* (2004 ▶, 2005 ▶); Aghabozorg, Motyeian *et al.* (2008 ▶); Aghabozorg, Derikvand *et al.* (2008 ▶). For the isotypic Co compound, see: Aghabozorg *et al.* (2009 ▶).
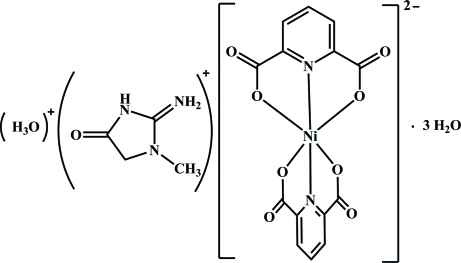


## Experimental

### 

#### Crystal data

(C_4_H_8_N_3_O)(H_3_O)[Ni(C_7_H_3_NO_4_)_2_]·3H_2_O
*M* *_r_* = 576.12
Triclinic, 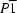

*a* = 8.1466 (9) Å
*b* = 10.7420 (12) Å
*c* = 13.5061 (15) Å
α = 74.890 (2)°
β = 89.944 (2)°
γ = 87.564 (3)°
*V* = 1140.0 (2) Å^3^
*Z* = 2
Mo *K*α radiation
μ = 0.93 mm^−1^
*T* = 120 K
0.18 × 0.14 × 0.12 mm

#### Data collection

Bruker SMART 1000 CCD area-detector diffractometer
Absorption correction: multi-scan (*SADABS*; Bruker, 1998 ▶) *T*_min_ = 0.803, *T*_max_ = 0.896
11509 measured reflections
5404 independent reflections
3827 reflections with *I* > 2σ(*I*)
*R*_int_ = 0.046

#### Refinement

*R*[*F* ^2^ > 2σ(*F* ^2^)] = 0.053
*wR*(*F* ^2^) = 0.134
*S* = 1.00
5404 reflections
335 parameters
H-atom parameters constrained
Δρ_max_ = 0.84 e Å^−3^
Δρ_min_ = −0.37 e Å^−3^

### 

Data collection: *SMART* (Bruker, 1998 ▶); cell refinement: *SAINT-Plus* (Bruker, 1998 ▶); data reduction: *SAINT-Plus*; program(s) used to solve structure: *SHELXS97* (Sheldrick, 2008 ▶); program(s) used to refine structure: *SHELXL97* (Sheldrick, 2008 ▶); molecular graphics: *SHELXTL* (Sheldrick, 2008 ▶); software used to prepare material for publication: *SHELXTL*.

## Supplementary Material

Crystal structure: contains datablocks I, global. DOI: 10.1107/S1600536809022053/pv2161sup1.cif

Structure factors: contains datablocks I. DOI: 10.1107/S1600536809022053/pv2161Isup2.hkl

Additional supplementary materials:  crystallographic information; 3D view; checkCIF report

## Figures and Tables

**Table 1:** Hydrogen-bond geometry (Å, °)

*D*—H⋯*A*	*D*—H	H⋯*A*	*D*⋯*A*	*D*—H⋯*A*
N3—H3*N*⋯O1*W*	0.85	1.89	2.726 (4)	166
N5—H5*NB*⋯O3^i^	0.93	1.96	2.861 (4)	162
N5—H5*NA*⋯O8^ii^	0.83	1.97	2.777 (4)	165
O1*W*—H1*WA*⋯O3*W*	0.85	1.84	2.662 (3)	162
O1*W*—H1*WB*⋯O7^ii^	0.85	1.97	2.804 (3)	166
O2*W*—H2*WA*⋯O6	0.85	1.71	2.529 (3)	161
O2*W*—H2*WB*⋯O4^iii^	0.85	1.65	2.480 (3)	165
O2*W*—H2*WC*⋯O4*W*	0.85	1.73	2.521 (3)	155
O3*W*—H3*WA*⋯O9	0.85	2.17	2.926 (4)	148
O3*W*—H3*WB*⋯O2^iv^	0.85	1.94	2.765 (4)	162
O4*W*—H4*WA*⋯O2^iv^	0.85	1.85	2.682 (4)	165
O4*W*—H4*WB*⋯O5^v^	0.85	1.91	2.722 (4)	159

Symmetry codes: (i) 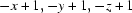; (ii) 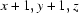; (iii) 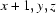; (iv) 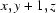; (v) 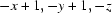.

**Fig. 1.**
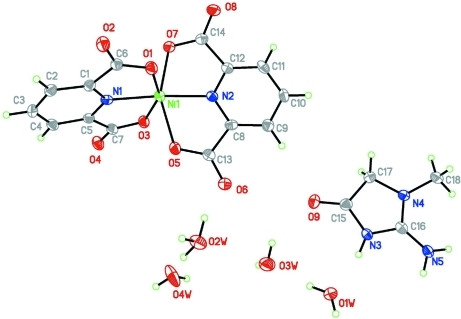
The molecular structure of the title compound, thermal ellipsoids are shown at 50% probability level.

**Fig. 2.**
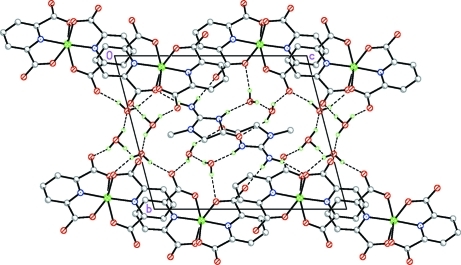
Unit cell packing diagram of the title compound, hydrogen bonds are shown as dashed lines and H-atoms not involved in H-bonds have been excluded.

**Fig. 3.**
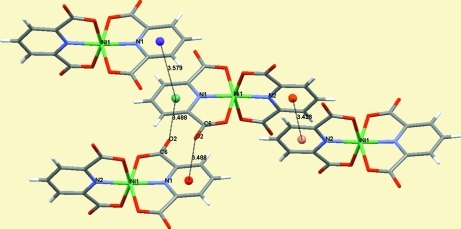
π-π Stacking interaction between two aromatic rings of (pydc)2– units.

## supplementary crystallographic information

### Comment

Two proton transfer compounds have been prepared from creatinine as proton acceptor agent by using pyridine-2,6-dicarboxylic acid (pydcH_2_) (Moghimi *et al.*, 2004) and 1,10-phenanthroline-9,13-dicarboxylic acid (phendcH_2_) (Soleimannejad *et al.*, 2005) as proton donor agents. Also three complexes of Bi^III^ (Aghabozorg, Ramezanipour *et al.*, 2008), Zn^II^ (Moghimi *et al.*, 2005) and Cr^III^ (Aghabozorg, Derikvand *et al.*, 2008) have been reported by our research group in which the (creatH)^+^ fragment was a part of the the crystal structure; more details can be found in our recently published review article (Aghabozorg, Manteghi *et al.*, 2008). We have now synthesized a novel Ni^II^ complex, (I), and determined its crystal structure which is presented in this article.

The crystal structure of the title complex, (I), (Fig. 1) contains [Ni(pydc)_2_]^2–^, creatininium (creatH)^+^ as counter-ion, hydroxonium cation, (H_3_O)^+^ and three uncoordinated water molecules. The Ni^II^ atom is coordinated by two tridentate (pydc)^2–^ groups and the presence of (creatH)^+^ and (H_3_O)^+^ ions balance the negative charge. In the structure of (I), the mean Ni—N and Ni—O bond distances are 1.973 (3) and 2.140 (2) Å, respectively, which are consistent with the corresponding distances reported for similar Ni^II^ complexes (Aghabozorg, Motyeian *et al.*, 2008). The N-atoms (N1 and N2) of the two (pydc)^2–^ fragments occupy the axial positions, while O1, O3, O5 and O7 atoms form the equatorial plane. In the anionic complex, the N1—Ni1—N2 angle [174.74 (11)°] deviates slightly from linearity. Therefore, the coordination around the Ni^II^ atom is distorted octahedral. In addition, the O1—Ni1—O3 and O5—Ni1—O7 angles [155.14 (9) and 155.22 (9)°] indicate that the four carboxylate groups of the two dianions are oriented in a flattened tetrahedral arrangement around the Ni^II^ atom. The angles O1—Ni1—O5, O1—Ni1—O7, O3—Ni1—O5 and O3—Ni1—O7 are 96.01 (9), 89.16 (9), 89.63 (9) and 95.81 (9)°, respectively. On the other hand, the torsion angles O1—Ni1—O7—C14 and O5—Ni1—O3—C7 are 93.3 (2) and 93.6 (2)°, respectively, indicating that two (pydc)^2–^ units are almost perpendicular to each other. The angle between two planes passing aromatic rings of (pydc)^2–^ units is 81.13 (15)°.

A remarkable feature of the title compound is the presence of a large number of O—H···O, N—H···O and C—H···O hydrogen bonds, with D···A distances ranging from 2.480 (3) to 3.415 (4) Å between (creatH)^+^, (H_3_O)^+^ and [Ni(pydc)_2_]^2–^ fragments and uncoordinated water molecules. The O2W—O2WB···O4^iii^ hydrogen bond with D···A distance of 2.480 (3) Å, is the strongest (details of the hydrogen bonding geometry have been provided in Table 1). These interactions link the fragments to form a three-dimensional network as shown in Fig. 2. In the structure of (I), the space between two layers of [Ni(pydc)_2_]^2–^ anions is filled with layers of (creatH)^+^, (H_3_O)^+^ cations and uncoordinated water molecules (Fig. 2).

There is noticeable π-π stacking interaction between two aromatic rings of (pydc)^2–^ units, with distances 3.428 (2) Å (1 - *x*, -*y*, -*z* + 1) and 3.579 (2) Å (-*x*, -*y*, -*z*). Also a considerable centrosymmetric C═O···π stacking interactions between C═O groups of carboxylate fragments with aromatic rings of pyridine-2,6-dicarboxylate with distance of 3.488 (1) Å for C═O2···*Cg*1 (1 - *x*, -*y*, -*z*) [*Cg*1 is the centroid for N1/C1—C5 ring] are observed in (I) (Fig. 3).

### Experimental

The reaction of nickel(II) chloride hexahydrate (59 mg, 0.25 mmol), creatinine, creat, (57 mg, 0.5 mmol) and pyridine-2,6-dicarboxylic acid, pydcH_2_, (83 mg, 0.5 mmol) in a 1:2:2 molar ratio in aqueous solution resulted in the formation of green, (H_3_O)(creatH)[Ni(pydc)_2_]. 3H_2_O crystals.

### Refinement

The H atoms of water molecules, NH and NH_2_ groups were located in difference Fourier synthesis. All hydrogen atoms were included in the refinement in isotropic approximatiom in riding model with distances: N—H (at positions from difference map), C—H = 0.95 (aryl), 0.98 (methyl), 0.99 (methylene) and O—H = 0.85 Å, and the *U*_iso_(H) parameters equal to 1.2 *U*_eq_(C), 1.5 *U*_eq_(O,*N*), where U(C,*O*,*N*) are respectively the equivalent thermal parameters of the C, O and N atoms to which corresponding H atoms were bonded.

### Crystal data

**Table:**

(C_4_H_8_N_3_O)(H_3_O)[Ni(C_7_H_3_NO_4_)_2_]·3H_2_O	*Z* = 2
*M**_r_* = 576.12	*F*(000) = 596
Triclinic, *P*1	*D*_x_ = 1.678 Mg m^−^^3^
Hall symbol: -P 1	Mo *K*α radiation, λ = 0.71073 Å
*a* = 8.1466 (9) Å	Cell parameters from 1838 reflections
*b* = 10.7420 (12) Å	θ = 2.8–24.2°
*c* = 13.5061 (15) Å	µ = 0.93 mm^−^^1^
α = 74.890 (2)°	*T* = 120 K
β = 89.944 (2)°	Prism, green
γ = 87.564 (3)°	0.18 × 0.14 × 0.12 mm
*V* = 1140.0 (2) Å^3^	

### Data collection

**Table:**

Bruker SMART 1000 CCD area-detector diffractometer	5404 independent reflections
Radiation source: fine-focus sealed tube	3827 reflections with *I* > 2σ(*I*)
graphite	*R*_int_ = 0.046
ω scans	θ_max_ = 28.0°, θ_min_ = 2.0°
Absorption correction: multi-scan (*SADABS*; Bruker, 1998)	*h* = −10→10
*T*_min_ = 0.803, *T*_max_ = 0.896	*k* = −14→14
11509 measured reflections	*l* = −17→17

### Refinement

**Table:**

Refinement on *F*^2^	Primary atom site location: structure-invariant direct methods
Least-squares matrix: full	Secondary atom site location: difference Fourier map
*R*[*F*^2^ > 2σ(*F*^2^)] = 0.053	Hydrogen site location: mixed
*wR*(*F*^2^) = 0.134	H-atom parameters constrained
*S* = 1.00	*w* = 1/[σ^2^(*F*_o_^2^) + (0.0405*P*)^2^ + 2.37*P*] where *P* = (*F*_o_^2^ + 2*F*_c_^2^)/3
5404 reflections	(Δ/σ)_max_ = 0.001
335 parameters	Δρ_max_ = 0.84 e Å^−^^3^
0 restraints	Δρ_min_ = −0.37 e Å^−^^3^

### Special details

**Table:**

Geometry. All e.s.d.'s (except the e.s.d. in the dihedral angle between two l.s. planes) are estimated using the full covariance matrix. The cell e.s.d.'s are taken into account individually in the estimation of e.s.d.'s in distances, angles and torsion angles; correlations between e.s.d.'s in cell parameters are only used when they are defined by crystal symmetry. An approximate (isotropic) treatment of cell e.s.d.'s is used for estimating e.s.d.'s involving l.s. planes.
Refinement. Refinement of *F*^2^ against ALL reflections. The weighted *R*-factor *wR* and goodness of fit *S* are based on *F*^2^, conventional *R*-factors *R* are based on *F*, with *F* set to zero for negative *F*^2^. The threshold expression of *F*^2^ > σ(*F*^2^) is used only for calculating *R*-factors(gt) *etc*. and is not relevant to the choice of reflections for refinement. *R*-factors based on *F*^2^ are statistically about twice as large as those based on *F*, and *R*- factors based on ALL data will be even larger.

### Fractional atomic coordinates and isotropic or equivalent isotropic displacement parameters (Å^2^)

**Table:**

	*x*	*y*	*z*	*U*_iso_*/*U*_eq_	
Ni1	0.26883 (5)	0.07342 (4)	0.22939 (3)	0.01905 (13)	
O1	0.4241 (3)	−0.0881 (2)	0.23014 (17)	0.0225 (5)	
O2	0.4819 (3)	−0.2302 (2)	0.13848 (18)	0.0245 (5)	
O3	0.0829 (3)	0.2229 (2)	0.16633 (17)	0.0211 (5)	
O4	−0.0572 (3)	0.3100 (2)	0.01983 (17)	0.0254 (5)	
O5	0.4573 (3)	0.2152 (2)	0.18788 (17)	0.0220 (5)	
O6	0.6101 (3)	0.3404 (2)	0.25748 (18)	0.0271 (5)	
O7	0.1100 (3)	−0.0493 (2)	0.33115 (17)	0.0228 (5)	
O8	0.0881 (3)	−0.1386 (2)	0.50037 (18)	0.0271 (5)	
N1	0.2282 (3)	0.0494 (2)	0.0918 (2)	0.0165 (5)	
N2	0.3261 (3)	0.1077 (2)	0.3616 (2)	0.0161 (5)	
C1	0.3098 (4)	−0.0458 (3)	0.0640 (2)	0.0175 (6)	
C2	0.2950 (4)	−0.0601 (3)	−0.0352 (2)	0.0201 (7)	
H2A	0.3515	−0.1291	−0.0547	0.024*	
C3	0.1952 (4)	0.0295 (3)	−0.1048 (3)	0.0221 (7)	
H3A	0.1842	0.0227	−0.1732	0.027*	
C4	0.1118 (4)	0.1285 (3)	−0.0749 (2)	0.0208 (7)	
H4A	0.0440	0.1904	−0.1221	0.025*	
C5	0.1297 (4)	0.1347 (3)	0.0252 (2)	0.0174 (6)	
C6	0.4140 (4)	−0.1286 (3)	0.1508 (2)	0.0189 (7)	
C7	0.0456 (4)	0.2315 (3)	0.0738 (2)	0.0203 (7)	
C8	0.4375 (4)	0.1947 (3)	0.3651 (2)	0.0175 (6)	
C9	0.4809 (4)	0.2192 (3)	0.4569 (2)	0.0207 (7)	
H9A	0.5607	0.2803	0.4591	0.025*	
C10	0.4039 (4)	0.1512 (3)	0.5470 (2)	0.0212 (7)	
H10A	0.4291	0.1671	0.6112	0.025*	
C11	0.2910 (4)	0.0610 (3)	0.5404 (2)	0.0220 (7)	
H11A	0.2383	0.0134	0.6003	0.026*	
C12	0.2554 (4)	0.0409 (3)	0.4455 (2)	0.0168 (6)	
C13	0.5092 (4)	0.2559 (3)	0.2620 (2)	0.0202 (7)	
C14	0.1407 (4)	−0.0569 (3)	0.4255 (2)	0.0193 (7)	
O9	0.7005 (3)	0.4732 (2)	0.46109 (18)	0.0300 (6)	
N3	0.8635 (3)	0.5880 (3)	0.5417 (2)	0.0186 (6)	
H3N	0.9098	0.6328	0.4886	0.028*	
N4	0.7837 (3)	0.5199 (3)	0.7029 (2)	0.0202 (6)	
N5	0.9931 (3)	0.6668 (3)	0.6669 (2)	0.0213 (6)	
H5NB	0.9862	0.6905	0.7286	0.032*	
H5NA	1.0388	0.7217	0.6217	0.032*	
C15	0.7458 (4)	0.5019 (3)	0.5378 (3)	0.0217 (7)	
C16	0.8858 (4)	0.5953 (3)	0.6403 (2)	0.0185 (6)	
C17	0.6828 (4)	0.4533 (3)	0.6458 (3)	0.0226 (7)	
H17A	0.5647	0.4771	0.6499	0.027*	
H17B	0.6996	0.3585	0.6710	0.027*	
C18	0.7939 (5)	0.4855 (4)	0.8144 (3)	0.0288 (8)	
H18A	0.8137	0.5627	0.8376	0.043*	
H18B	0.8844	0.4214	0.8376	0.043*	
H18C	0.6904	0.4491	0.8429	0.043*	
O1W	0.9969 (3)	0.7014 (2)	0.35667 (18)	0.0257 (5)	
H1WA	0.9086	0.6978	0.3243	0.038*	
H1WB	1.0206	0.7804	0.3398	0.038*	
O2W	0.7807 (3)	0.4512 (2)	0.10606 (19)	0.0310 (6)	
H2WA	0.7267	0.3996	0.1517	0.046*	
H2WB	0.8208	0.4006	0.0723	0.046*	
H2WC	0.7360	0.5102	0.0580	0.046*	
O3W	0.7459 (3)	0.6405 (3)	0.2550 (2)	0.0385 (7)	
H3WA	0.7188	0.5734	0.3000	0.058*	
H3WB	0.6564	0.6811	0.2316	0.058*	
O4W	0.6023 (4)	0.6403 (3)	0.0074 (2)	0.0454 (8)	
H4WA	0.5592	0.6919	0.0393	0.068*	
H4WB	0.5964	0.6694	−0.0574	0.068*	

### Atomic displacement parameters (Å^2^)

**Table:**

	*U*^11^	*U*^22^	*U*^33^	*U*^12^	*U*^13^	*U*^23^
Ni1	0.0227 (2)	0.0187 (2)	0.0156 (2)	−0.00178 (16)	−0.00045 (16)	−0.00419 (16)
O1	0.0295 (13)	0.0209 (12)	0.0176 (11)	0.0012 (10)	0.0011 (10)	−0.0064 (9)
O2	0.0315 (13)	0.0208 (12)	0.0220 (12)	0.0086 (10)	−0.0026 (10)	−0.0087 (10)
O3	0.0254 (12)	0.0215 (12)	0.0168 (11)	0.0008 (10)	0.0001 (9)	−0.0062 (9)
O4	0.0287 (13)	0.0262 (13)	0.0193 (12)	0.0074 (10)	−0.0034 (10)	−0.0040 (10)
O5	0.0249 (12)	0.0263 (12)	0.0155 (11)	−0.0045 (10)	0.0021 (9)	−0.0060 (9)
O6	0.0306 (14)	0.0279 (13)	0.0220 (12)	−0.0110 (11)	0.0015 (10)	−0.0038 (10)
O7	0.0294 (13)	0.0204 (12)	0.0186 (12)	−0.0068 (10)	0.0023 (10)	−0.0042 (9)
O8	0.0349 (14)	0.0231 (12)	0.0222 (12)	−0.0078 (11)	0.0060 (10)	−0.0027 (10)
N1	0.0151 (13)	0.0140 (12)	0.0186 (13)	−0.0019 (10)	0.0003 (10)	−0.0009 (10)
N2	0.0170 (13)	0.0132 (12)	0.0167 (13)	0.0031 (10)	0.0000 (10)	−0.0023 (10)
C1	0.0153 (15)	0.0184 (15)	0.0193 (16)	−0.0025 (12)	0.0014 (12)	−0.0058 (12)
C2	0.0194 (16)	0.0195 (16)	0.0226 (17)	−0.0040 (13)	0.0014 (13)	−0.0071 (13)
C3	0.0268 (18)	0.0234 (17)	0.0170 (16)	−0.0025 (14)	−0.0039 (13)	−0.0065 (13)
C4	0.0216 (17)	0.0204 (16)	0.0178 (16)	−0.0010 (13)	−0.0030 (13)	−0.0002 (12)
C5	0.0180 (16)	0.0158 (15)	0.0178 (15)	−0.0031 (12)	0.0026 (12)	−0.0031 (12)
C6	0.0182 (16)	0.0195 (16)	0.0187 (16)	−0.0032 (13)	0.0012 (12)	−0.0039 (13)
C7	0.0229 (17)	0.0175 (15)	0.0188 (16)	0.0005 (13)	−0.0011 (13)	−0.0019 (12)
C8	0.0146 (15)	0.0129 (14)	0.0233 (16)	0.0017 (12)	0.0007 (12)	−0.0021 (12)
C9	0.0204 (16)	0.0193 (16)	0.0216 (16)	0.0027 (13)	−0.0028 (13)	−0.0046 (13)
C10	0.0263 (18)	0.0221 (16)	0.0157 (15)	0.0054 (14)	−0.0040 (13)	−0.0066 (13)
C11	0.0242 (17)	0.0226 (17)	0.0164 (16)	0.0071 (14)	0.0006 (13)	−0.0012 (13)
C12	0.0148 (15)	0.0151 (15)	0.0193 (16)	0.0040 (12)	0.0008 (12)	−0.0032 (12)
C13	0.0204 (16)	0.0196 (16)	0.0195 (16)	0.0003 (13)	0.0004 (13)	−0.0030 (13)
C14	0.0180 (16)	0.0168 (15)	0.0232 (17)	0.0015 (12)	0.0016 (13)	−0.0055 (13)
O9	0.0341 (14)	0.0351 (14)	0.0227 (13)	−0.0115 (12)	−0.0032 (11)	−0.0094 (11)
N3	0.0211 (14)	0.0209 (14)	0.0132 (13)	−0.0024 (11)	0.0022 (10)	−0.0035 (10)
N4	0.0246 (15)	0.0185 (13)	0.0165 (13)	−0.0040 (11)	0.0016 (11)	−0.0021 (11)
N5	0.0267 (15)	0.0225 (14)	0.0140 (13)	−0.0018 (12)	−0.0001 (11)	−0.0034 (11)
C15	0.0211 (16)	0.0188 (16)	0.0241 (17)	−0.0007 (13)	−0.0012 (13)	−0.0037 (13)
C16	0.0199 (16)	0.0165 (15)	0.0179 (16)	0.0050 (12)	0.0009 (12)	−0.0032 (12)
C17	0.0220 (17)	0.0231 (17)	0.0220 (17)	−0.0050 (14)	−0.0002 (13)	−0.0043 (13)
C18	0.040 (2)	0.0268 (18)	0.0181 (17)	−0.0089 (16)	0.0017 (15)	−0.0015 (14)
O1W	0.0261 (13)	0.0248 (13)	0.0248 (13)	−0.0066 (10)	−0.0014 (10)	−0.0034 (10)
O2W	0.0373 (15)	0.0255 (13)	0.0266 (13)	0.0023 (11)	0.0072 (11)	−0.0013 (11)
O3W	0.0363 (16)	0.0365 (15)	0.0345 (15)	−0.0107 (12)	−0.0101 (12)	0.0067 (12)
O4W	0.082 (2)	0.0346 (15)	0.0169 (13)	0.0270 (15)	−0.0012 (13)	−0.0065 (11)

### Geometric parameters (Å, °)

**Table:**

Ni1—N1	1.972 (3)	C10—C11	1.384 (5)
Ni1—N2	1.974 (3)	C10—H10A	0.9500
Ni1—O1	2.101 (2)	C11—C12	1.387 (4)
Ni1—O7	2.121 (2)	C11—H11A	0.9500
Ni1—O3	2.163 (2)	C12—C14	1.511 (4)
Ni1—O5	2.178 (2)	O9—C15	1.218 (4)
O1—C6	1.262 (4)	N3—C16	1.368 (4)
O2—C6	1.252 (4)	N3—C15	1.370 (4)
O3—C7	1.265 (4)	N3—H3N	0.8511
O4—C7	1.251 (4)	N4—C16	1.327 (4)
O5—C13	1.272 (4)	N4—C18	1.455 (4)
O6—C13	1.240 (4)	N4—C17	1.457 (4)
O7—C14	1.280 (4)	N5—C16	1.298 (4)
O8—C14	1.243 (4)	N5—H5NB	0.9334
N1—C1	1.330 (4)	N5—H5NA	0.8300
N1—C5	1.342 (4)	C15—C17	1.512 (5)
N2—C12	1.320 (4)	C17—H17A	0.9900
N2—C8	1.340 (4)	C17—H17B	0.9900
C1—C2	1.394 (4)	C18—H18A	0.9800
C1—C6	1.509 (4)	C18—H18B	0.9800
C2—C3	1.391 (5)	C18—H18C	0.9800
C2—H2A	0.9500	O1W—H1WA	0.8500
C3—C4	1.385 (5)	O1W—H1WB	0.8500
C3—H3A	0.9500	O2W—H2WA	0.8499
C4—C5	1.379 (4)	O2W—H2WB	0.8500
C4—H4A	0.9500	O2W—H2WC	0.8500
C5—C7	1.509 (4)	O3W—H3WA	0.8500
C8—C9	1.382 (5)	O3W—H3WB	0.8500
C8—C13	1.505 (4)	O4W—H4WA	0.8500
C9—C10	1.408 (5)	O4W—H4WB	0.8500
C9—H9A	0.9500		
			
N1—Ni1—N2	174.74 (11)	C8—C9—H9A	120.8
N1—Ni1—O1	78.05 (10)	C10—C9—H9A	120.8
N2—Ni1—O1	101.80 (10)	C11—C10—C9	118.8 (3)
N1—Ni1—O7	106.74 (10)	C11—C10—H10A	120.6
N2—Ni1—O7	78.49 (10)	C9—C10—H10A	120.6
O1—Ni1—O7	89.16 (9)	C10—C11—C12	119.3 (3)
N1—Ni1—O3	77.19 (9)	C10—C11—H11A	120.3
N2—Ni1—O3	103.06 (9)	C12—C11—H11A	120.3
O1—Ni1—O3	155.14 (9)	N2—C12—C11	121.0 (3)
O7—Ni1—O3	95.81 (9)	N2—C12—C14	112.9 (3)
N1—Ni1—O5	98.03 (9)	C11—C12—C14	126.1 (3)
N2—Ni1—O5	76.74 (10)	O6—C13—O5	126.9 (3)
O1—Ni1—O5	96.01 (9)	O6—C13—C8	117.6 (3)
O7—Ni1—O5	155.22 (9)	O5—C13—C8	115.5 (3)
O3—Ni1—O5	89.63 (9)	O8—C14—O7	125.9 (3)
C6—O1—Ni1	114.6 (2)	O8—C14—C12	118.2 (3)
C7—O3—Ni1	113.8 (2)	O7—C14—C12	115.9 (3)
C13—O5—Ni1	114.4 (2)	C16—N3—C15	110.3 (3)
C14—O7—Ni1	112.9 (2)	C16—N3—H3N	126.5
C1—N1—C5	121.1 (3)	C15—N3—H3N	123.1
C1—N1—Ni1	118.9 (2)	C16—N4—C18	125.2 (3)
C5—N1—Ni1	119.8 (2)	C16—N4—C17	110.3 (3)
C12—N2—C8	121.4 (3)	C18—N4—C17	123.3 (3)
C12—N2—Ni1	118.5 (2)	C16—N5—H5NB	122.1
C8—N2—Ni1	120.1 (2)	C16—N5—H5NA	119.2
N1—C1—C2	120.9 (3)	H5NB—N5—H5NA	111.9
N1—C1—C6	111.8 (3)	O9—C15—N3	125.8 (3)
C2—C1—C6	127.3 (3)	O9—C15—C17	127.5 (3)
C3—C2—C1	118.1 (3)	N3—C15—C17	106.7 (3)
C3—C2—H2A	120.9	N5—C16—N4	125.9 (3)
C1—C2—H2A	120.9	N5—C16—N3	123.6 (3)
C4—C3—C2	120.3 (3)	N4—C16—N3	110.6 (3)
C4—C3—H3A	119.8	N4—C17—C15	102.1 (3)
C2—C3—H3A	119.8	N4—C17—H17A	111.3
C5—C4—C3	118.3 (3)	C15—C17—H17A	111.3
C5—C4—H4A	120.9	N4—C17—H17B	111.3
C3—C4—H4A	120.9	C15—C17—H17B	111.3
N1—C5—C4	121.3 (3)	H17A—C17—H17B	109.2
N1—C5—C7	112.1 (3)	N4—C18—H18A	109.5
C4—C5—C7	126.6 (3)	N4—C18—H18B	109.5
O2—C6—O1	125.8 (3)	H18A—C18—H18B	109.5
O2—C6—C1	118.1 (3)	N4—C18—H18C	109.5
O1—C6—C1	116.1 (3)	H18A—C18—H18C	109.5
O4—C7—O3	126.4 (3)	H18B—C18—H18C	109.5
O4—C7—C5	117.0 (3)	H1WA—O1W—H1WB	105.5
O3—C7—C5	116.6 (3)	H2WA—O2W—H2WB	101.4
N2—C8—C9	121.1 (3)	H2WA—O2W—H2WC	123.5
N2—C8—C13	113.2 (3)	H2WB—O2W—H2WC	100.7
C9—C8—C13	125.7 (3)	H3WA—O3W—H3WB	106.0
C8—C9—C10	118.4 (3)	H4WA—O4W—H4WB	113.1
			
N1—Ni1—O1—C6	−3.8 (2)	Ni1—O1—C6—O2	−172.4 (3)
N2—Ni1—O1—C6	−178.4 (2)	Ni1—O1—C6—C1	7.4 (3)
O7—Ni1—O1—C6	103.5 (2)	N1—C1—C6—O2	171.8 (3)
O3—Ni1—O1—C6	1.4 (4)	C2—C1—C6—O2	−8.7 (5)
O5—Ni1—O1—C6	−100.8 (2)	N1—C1—C6—O1	−8.1 (4)
N1—Ni1—O3—C7	−4.7 (2)	C2—C1—C6—O1	171.5 (3)
N2—Ni1—O3—C7	169.9 (2)	Ni1—O3—C7—O4	−179.7 (3)
O1—Ni1—O3—C7	−10.0 (4)	Ni1—O3—C7—C5	2.4 (3)
O7—Ni1—O3—C7	−110.6 (2)	N1—C5—C7—O4	−175.4 (3)
O5—Ni1—O3—C7	93.6 (2)	C4—C5—C7—O4	4.0 (5)
N1—Ni1—O5—C13	178.0 (2)	N1—C5—C7—O3	2.7 (4)
N2—Ni1—O5—C13	−2.5 (2)	C4—C5—C7—O3	−177.9 (3)
O1—Ni1—O5—C13	−103.3 (2)	C12—N2—C8—C9	−1.0 (4)
O7—Ni1—O5—C13	−2.2 (4)	Ni1—N2—C8—C9	−179.2 (2)
O3—Ni1—O5—C13	101.0 (2)	C12—N2—C8—C13	177.3 (3)
N1—Ni1—O7—C14	170.5 (2)	Ni1—N2—C8—C13	−1.0 (3)
N2—Ni1—O7—C14	−8.9 (2)	N2—C8—C9—C10	−0.6 (5)
O1—Ni1—O7—C14	93.3 (2)	C13—C8—C9—C10	−178.7 (3)
O3—Ni1—O7—C14	−111.1 (2)	C8—C9—C10—C11	1.4 (5)
O5—Ni1—O7—C14	−9.3 (4)	C9—C10—C11—C12	−0.7 (5)
O1—Ni1—N1—C1	−1.0 (2)	C8—N2—C12—C11	1.8 (4)
O7—Ni1—N1—C1	−86.4 (2)	Ni1—N2—C12—C11	−179.9 (2)
O3—Ni1—N1—C1	−178.7 (2)	C8—N2—C12—C14	−176.9 (3)
O5—Ni1—N1—C1	93.5 (2)	Ni1—N2—C12—C14	1.4 (3)
O1—Ni1—N1—C5	−175.6 (2)	C10—C11—C12—N2	−0.9 (5)
O7—Ni1—N1—C5	98.9 (2)	C10—C11—C12—C14	177.5 (3)
O3—Ni1—N1—C5	6.6 (2)	Ni1—O5—C13—O6	−176.5 (3)
O5—Ni1—N1—C5	−81.1 (2)	Ni1—O5—C13—C8	2.8 (3)
O1—Ni1—N2—C12	−83.0 (2)	N2—C8—C13—O6	178.0 (3)
O7—Ni1—N2—C12	3.6 (2)	C9—C8—C13—O6	−3.9 (5)
O3—Ni1—N2—C12	97.0 (2)	N2—C8—C13—O5	−1.3 (4)
O5—Ni1—N2—C12	−176.5 (2)	C9—C8—C13—O5	176.8 (3)
O1—Ni1—N2—C8	95.3 (2)	Ni1—O7—C14—O8	−165.9 (3)
O7—Ni1—N2—C8	−178.0 (2)	Ni1—O7—C14—C12	12.2 (3)
O3—Ni1—N2—C8	−84.7 (2)	N2—C12—C14—O8	168.8 (3)
O5—Ni1—N2—C8	1.8 (2)	C11—C12—C14—O8	−9.8 (5)
C5—N1—C1—C2	−0.3 (4)	N2—C12—C14—O7	−9.5 (4)
Ni1—N1—C1—C2	−174.8 (2)	C11—C12—C14—O7	171.9 (3)
C5—N1—C1—C6	179.3 (3)	C16—N3—C15—O9	−178.9 (3)
Ni1—N1—C1—C6	4.8 (3)	C16—N3—C15—C17	2.2 (4)
N1—C1—C2—C3	1.3 (5)	C18—N4—C16—N5	−11.9 (5)
C6—C1—C2—C3	−178.2 (3)	C17—N4—C16—N5	−179.3 (3)
C1—C2—C3—C4	−0.9 (5)	C18—N4—C16—N3	167.9 (3)
C2—C3—C4—C5	−0.5 (5)	C17—N4—C16—N3	0.5 (4)
C1—N1—C5—C4	−1.2 (5)	C15—N3—C16—N5	178.1 (3)
Ni1—N1—C5—C4	173.3 (2)	C15—N3—C16—N4	−1.8 (4)
C1—N1—C5—C7	178.2 (3)	C16—N4—C17—C15	0.8 (3)
Ni1—N1—C5—C7	−7.3 (3)	C18—N4—C17—C15	−166.9 (3)
C3—C4—C5—N1	1.6 (5)	O9—C15—C17—N4	179.4 (3)
C3—C4—C5—C7	−177.8 (3)	N3—C15—C17—N4	−1.8 (3)

### Hydrogen-bond geometry (Å, °)

**Table:**

*D*—H···*A*	*D*—H	H···*A*	*D*···*A*	*D*—H···*A*
N3—H3N···O1W	0.85	1.89	2.726 (4)	166
N5—H5NB···O3^i^	0.93	1.96	2.861 (4)	162
N5—H5NA···O8^ii^	0.83	1.97	2.777 (4)	165
O1W—H1WA···O3W	0.85	1.84	2.662 (3)	162
O1W—H1WB···O7^ii^	0.85	1.97	2.804 (3)	166
O2W—H2WA···O6	0.85	1.71	2.529 (3)	161
O2W—H2WB···O4^iii^	0.85	1.65	2.480 (3)	165
O2W—H2WC···O4W	0.85	1.73	2.521 (3)	155
O3W—H3WA···O9	0.85	2.17	2.926 (4)	148
O3W—H3WB···O2^iv^	0.85	1.94	2.765 (4)	162
O4W—H4WA···O2^iv^	0.85	1.85	2.682 (4)	165
O4W—H4WB···O5^v^	0.85	1.91	2.722 (4)	159

Symmetry codes:  (i) −*x*+1, −*y*+1, −*z*+1; (ii) *x*+1, *y*+1, *z*; (iii) *x*+1, *y*, *z*; (iv) *x*, *y*+1, *z*; (v) −*x*+1, −*y*+1, −*z*.
